# ChemR23 signaling ameliorates brain injury via inhibiting NLRP3 inflammasome-mediated neuronal pyroptosis in ischemic stroke

**DOI:** 10.1186/s12967-023-04813-0

**Published:** 2024-01-04

**Authors:** Lan Liu, Jiawei Zhang, Kaili Lu, Yaxuan Zhang, Xiaofeng Xu, Jiangshan Deng, Xiaojie Zhang, Haibing Zhang, Yuwu Zhao, Xiuzhe Wang

**Affiliations:** 1 Department of Neurology, Shanghai Sixth People’s Hospital, Affiliated to Shanghai Jiao Tong University School of Medicine, 600 Yishan Road, Shanghai, 200233 China; 2 CAS Key Laboratory of Nutrition, Metabolism and Food Safety, Shanghai Institute of Nutrition and Health, University of Chinese Academy of Sciences, Chinese Academy of Sciences, Shanghai, China; 3 Shanghai Neurological Rare Disease Biobank and Precision Diagnostic Technical Service Platform, Shanghai, China

**Keywords:** Stroke, ChemR23, Pyroptosis, NLRP3 inflammasome, Neuron

## Abstract

**Background:**

Inflammatory response has been recognized as a pivotal pathophysiological process during cerebral ischemia. ChemR23 signaling is involved in the pathophysiology of various inflammatory diseases. Nevertheless, the role of ChemR23 signaling in ischemic stroke remains largely unknown.

**Methods:**

Permanent ischemic stroke mouse model was accomplished by middle cerebral artery occlusion (MCAO). Resolvin E1 (RvE1) or chemerin-9 (C-9), the agonists of ChemR23, were administered by intracerebroventricular (i.c.v) injection before MCAO induction. Then, analysis of neurobehavioral deficits and brain sampling were done at Day 1 after MCAO. The brain samples were further analyzed by histological staining, immunofluorescence, RNA sequencing, ELISA, transmission electron microscope, and western blots**.** Furthermore, oxygen–glucose deprivation (OGD) was employed in SH-SY5Y to mimic MCAO in vitro, and ChemR23 signaling pathway was further studied by overexpression of ChemR23 or administration of related agonists or antagonists. Analysis of cell death and related pathway markers were performed.

**Results:**

ChemR23 expression was upregulated following MCAO. Under in vitro and in vivo ischemic conditions, ChemR23 deficiency or inhibition contributed to excessive NLRP3-mediated maturation and release of IL-1β and IL-18, as well as enhanced cleavage of GSDMD-N and neuronal pyroptosis. These influences ultimately aggravated brain injury and neuronal damage. On the other hand, ChemR23 activation by RvE1 or C-9 mitigated the above pathophysiological abnormalities in vivo and in vitro, and overexpression of ChemR23 in SH-SY5Y cells also rescued OGD-induced neuronal pyroptosis. Blockade of NLRP3 mimics the protective effects of ChemR23 activation in vitro.

**Conclusion:**

Our data indicated that ChemR23 modulates NLRP3 inflammasome-mediated neuronal pyroptosis in ischemic stroke. Activation of ChemR23 may serve as a promising potential target for neuroprotection in cerebral ischemia.

**Supplementary Information:**

The online version contains supplementary material available at 10.1186/s12967-023-04813-0.

## Introduction

Ischemic stroke is a common cerebrovascular disease caused by a sudden reduction or blockage of cerebral blood flow. It’s the main cause of disability and the leading public health threat worldwide [1]. Post-ischemic inflammatory response begins within several minutes after the onset of brain ischemia. Secondary neuroinflammation promotes further injury and has a great impact on the outcome of stroke [2]. Nowadays, the molecular mechanisms underlying inflammation-induced neuronal cell death in ischemic stroke are complex and remain to be fully understood.

Inflammasome-mediated cell death, namely pyroptosis, is identified as an important mechanism of inflammation-induced neuronal cell death in ischemic stroke [3]. Accumulating evidence suggests that NOD-leucine rich repeat and pyrin containing protein 3 (NLRP3) inflammasomes are important drivers of the caspase-1 cleavage [4, 5]. Activated caspase-1 promotes the maturation and release of pro-inflammatory interleukin (IL)-1β and IL-18 [3], which subsequently lead to the cleavage of gasdermin D (GSDMD) [6]. The N-terminal fragment of GSDMD binds to phosphatidylserine and cardiolipin on cell membrane, forms membrane pores, and ultimately elicits a type of programmed cell death known as pyroptosis [7]. A number of studies have shown that NLRP3 could be triggered by ischemic stroke and aggravate brain injury [8, 9]. Thus, targeting NLRP3 inflammasome-mediated neuronal pyroptosis may provide new insight and a theoretical basis for developing an effective treatment for ischemic stroke.

The chemokine-like receptor 1 (CMKLR1 or ChemR23, and the later nomenclature is used in this study) is a seven-pass transmembrane G protein-coupled receptor for the chemoattractant adipokine chemerin and the pro-resolving molecule, resolvin E1 (RvE1). ChemR23 is abundantly expressed on peripheral immune cells such as macrophages and dendritic cells [10], as well as neurons and glial cells in the central nervous system (CNS) [11]. RvE1 and chemerin are endogenous ligands of ChemR23 and play an important role in regulating inflammation [12, 13]. It has been reported that ChemR23 signaling is involved in peripheral ischemia diseases. For example, early treatment with RvE1 could suppress the infiltration of dominant Ly6^Chi^ monocyte/macrophage and secretion of pro-inflammatory cytokines in acute myocardial infarction, and improve cardiac function by activating ChemR23 [14]. Furthermore, chemerin/ChemR23 axis could regulate normal angiogenesis and hypoxia‑driven neovascularization in the hind-limb ischemia model [15]. To date, knowledge about the functions of ChemR23 signaling in the brain is limited. RvE1 and chemerin have shown beneficial effects in models of neonatal germinal matrix hemorrhage, neonatal hypoxic–ischemic encephalopathy, chronic neuropathic pain, and Alzheimer’s disease [16–19]. However, the role of ChemR23 signaling in ischemic stroke remains elusive.

In this study, we investigated the functions of ChemR23 in cerebral ischemia by utilizing middle cerebral artery occlusion (MCAO) mouse model and neuronal oxygen–glucose deprivation (OGD) model.

## Materials and methods

### Animals and ischemic stroke animal model

Wild-type (WT) male C57BL/6 mice and ChemR23^−/−^ mice were purchased from the Shanghai Model Organisms Center (Shanghai, China). Mice were housed in a climate-controlled room, maintained on a 12-h light/dark cycle with water and food available ad libitum. All procedures were approved by the ethical committee on animal welfare of Sixth People’s Hospital Affiliated to Shanghai Jiao Tong University School of Medicine and were performed in conformity with the National Institute of Health Guide for the Care and Use of Laboratory Animals (NIH Publications No. 8023, revised 2022).

The middle cerebral artery occlusion model (MCAO) model was produced as described previously [8]. Briefly, animals were anesthetized with 1% pentobarbital sodium (10 mg/kg). After the isolation of left common carotid artery (CCA), external and internal carotid arteries (ECA and ICA), a monofilament nylon filament with a diameter of 0.16 mm, 25 mm in length (Beijing Cinontech Biotech Co. Ltd, Beijing, China) coated with silicon hardener mixture (a diameter of 0.20 ± 0.01 mm) was inserted into the ECA stump, then reversed into the ICA and finally to the left ostium of the MCA (near 10 mm). The success of occlusion was characterized by the reduction in MCA cortical blood flow down to 20% of the baseline, which was monitored by a laser Doppler flowmetry (Moor Instruments, Devon, England). Sham-operated mice underwent the same procedure without inserting the monofilament nylon filament. Body temperature was regularly maintained at 37 °C throughout the surgical procedure, using a thermoregulated heating pad (RWD Life Science, Shenzhen, China). After the surgery, animals were placed at room temperature and returned to the cage until awake.

### Drug administration

Different dosages of RvE1 (0.3 and 1.0 nmol, No. 10007848, Cayman Chemical Company, USA) and Chemerin-9 (C-9) (3, 9, and 27 μg/kg, No. 7117, Tocris Bioscience, Bristol, UK) were intracerebroventricularly (i.c.v) injected into the left lateral ventricle LV (AP = − 0.2 mm, ML = − 1 mm, DV = − 2.5 mm) stereotaxically 30 min before surgery. The doses of RvE1 and C-9 were referred to previously published studies, respectively [17, 20]. The control mice were stereotaxically injected with equivalent phosphate-buffered saline (PBS) as a vehicle.

### Study protocol

A total of 262 male mice (20–25 g, 6–8 weeks) were analyzed in this study. All mice were randomly divided into three different experiments (Additional file 1: Fig. S1).

Experiment 1: To explore the time course of dynamic changes in ChemR23 expression in ipsilateral hemisphere, mice were randomly divided into 5 groups (Sham, 6 h, 12 h, 1 day, and 3 days after MCAO). Western blot (n = 3/group) and qRT-PCR (n = 3/group) analyses were performed to detect the expression of ChemR23 in the ipsilateral brain of mice after MCAO.

Experiment 2: To elucidate the effects of ChemR23 signaling in ischemic stroke, ChemR23^−/−^ mice (KO) were employed. Mice were randomly assigned to four groups: WT + Sham, WT + MCAO, KO + Sham, and KO + MCAO. Behavioral tests (n = 10/group) and infarct volume (n = 6/group) were performed on Day 1, Day 3, Day 7, and Day 14 after MCAO. And RNA sequencing (n = 4/group), immunofluorescence (n = 3/group), enzyme-linked immunosorbent assay (ELISA) analysis (n = 4/group), western blot (n = 3/group), and transmission electron microscope (TEM) (n = 3/group) were evaluated at Day 1 post-modeling.

Experiment 3: To investigate the effect of ChemR23 activation by RvE1 or C-9, mice were randomly divided into the following groups: WT + MCAO + PBS, WT + MCAO + RvE1, WT + MCAO + C-9, KO + MCAO + PBS, KO + MCAO + RvE1, and KO + MCAO + C-9. Behavioral tests (n = 10/group), infarct volume (n = 6/group), immunofluorescence (n = 3/group), ELISA analysis (n = 4), western blot (n = 3/group), and transmission electron microscope (TEM) (n = 3/group) were evaluated at Day 1 after treatment.

### Behavioral tests

Behavioral tests were performed prior to MCAO and at Day 1, Day 3, Day 7, and Day 14 after MCAO. Mice were trained for 3 days before surgery, and the baseline neurological function values were generated simultaneously by averaging 3 trials before MCAO.

The modified neurologic severity scores (mNSS), ranging from 0 to 14 (normal to maximal deficit score of 14), is a scale to evaluate the neurological function, including motor (muscle status, abnormal movement), sensation (visual, tactile, and proprioceptive), balance, and reflex abilities testing items [8]. One score point is recorded for the incapability to accomplish one of the tests or for the lack of a tested reflex. Thus, higher scores indicate more severe neurological impairments.

Grip strength test was measured using a grip-force measurement system (No. SA417, SANS, China). In this test, a wire mesh screen measuring 10 cm × 12 cm was coupled to a dynamometer that recorded the force of the animal’s paws (either the forelimbs or hindlimbs). The tail was gently and steadily pulled to measure the maximum force until the mouse released the wire mesh screen. The grip strength meter software collected the peak grip force produced in gram-force (g) to release the wire mesh screen. Each animal was tested 3 times with a 2–3 min interval to obtain mean peak force [21].

### Infarct volume measurement

The 2, 3, 5-Tripthenyltetrazolum Chloride (TTC, Sigma-Aldrich, St Louis, USA) staining in the acute phase (Day 1 and Day 3) of cerebral infarction and Cresyl Violet (Sigma-Aldrich, St Louis, USA) staining in the chronic phase (Day 7 and Day 14) were performed to evaluate infarct size. In brief, the mouse brains were immediately removed after sacrifice and cut into sections coronally. For TTC staining, the brains were cut into 2 mm-thick sections, and then the slices were stained with 2% TTC at 37 °C for 20 min. For Cresyl Violet staining, a series of 20-μm-thick slices with a 300-μm interval from anterior commissure to hippocampus were stained by 0.1% Cresyl Violet at 37 °C for 5 min, and then fixed in 4% paraformaldehyde (PFA) overnight at 4℃. Infarct volume was measured by ImageJ software (National Institutes of Health). The infarct ratio was calculated by the following formula: corrected infarct volume (%) = (contralateral hemispheric volume—ipsilateral non-infarcted volume)/contralateral hemispheric volume × 100%.

### RNA sequencing (RNA-seq)

The ipsilateral hemispheres of MCAO at Day 1were randomly collected from four mice in each group and processed for RNA extraction, cDNA library construction and RNA-seq by Xuran Biotechnology (Shanghai, China). Corrected p-value of 0.05 and log 2 (fold change) of 1 were set as the threshold for significant differential expressed genes (DEGs). The DEGs of each group were subjected to the Gene Ontology (GO) and Kyoto Encyclopedia of Genes and Genomes (KEGG) pathway enrichment functional analysis.

### TUNEL staining

To detect neuronal cell death, double immunostaining of NeuN and terminal deoxynucleotidyl transferase dUTP nick end labeling (TUNEL) was carried out using One Step TUNEL Assay Kit (Beyotime, China). Briefly, brain slices were incubated with anti-NeuN (ab177487, 1:200, Abcam, USA) overnight at 4 °C, and then immersed in TUNEL mixture at room temperature for 1 h, followed by DAPI staining. The TUNEL positive neurons were counted in 3 microscopic fields at 400 × magnification at the left, right, and bottom of peri-infarct area in each mouse by a blinded observer. Data were expressed as the ratio of TUNEL positive neurons (relative to Sham group).

### Immunofluorescence staining and quantification

Immediately after behavioral tests, mice were transcardially perfused with 0.9% NaCl and 4% ice-cold PFA. Subsequently, PFA-fixed brain samples were embedded in paraffin and then sliced into coronal sections in 4 µm thickness with microtome. For immunofluorescent staining, paraffin slices were deparaffinized, rehydrated, and subjected to heat-induced antigen retrieval using a microwave. Subsequently, brain sections and treated cell coverslips were fixed with 4% PFA for 20 min and immersed in blocking buffer (3% donkey serum and 0.3% Triton X-100 in PBS) at room temperature for 1 h, and then incubated with primary antibodies at 4 °C overnight. The following primary antibodies were used: anti-NLRP3 (rabbit, BA3677, 1:200, Boster, China), anti-GSDMD (rabbit, A22602, 1:200, Abclonal, China) and anti-NeuN (goat, 1:500, Abcam, USA). After 3 times of rinsing by PBS for 10 min, sections were then incubated with fluorescence-conjugated secondary antibodies, including donkey anti-rabbit secondary antibody (Alexa Fluor 488^®^, 1:500, CST, USA) or donkey anti-goat secondary antibody (Alexa Fluor 594^®^, 1:500, CST, USA) for 1 h at room temperature. After washing with PBS, sections were counterstained with 4′,6-diamidino-2-phenylindole dihydrochloride (DAPI, Beyotime, China) followed by covering with anti-quenching fluorescence mounting medium. Subsequently, the sections were visualized under a microscope (IX53, Olympus, Tokyo, Japan). Appropriate positive and negative controls were performed for each batch of slides. The number of NLRP3 positive neurons and GSDMD positive neurons were analyzed in the same manner as for TUNEL staining.

### Transmission electron microscope (TEM)

Peri-infarct tissue (1 × 1 × 1 mm) samples were immersed in 2.5% glutaraldehyde for 1 day and then fixed in 1% osmium tetroxide for 3 h. After dehydrating in ethanol (50%, 70%, 90%, and 100%) for 10 min each and embedded in araldite, tissues were cut into a thickness of 70 nm. Finally, the sections were stained with 3% uranyl acetate and lead citrate and scanned using a Transmission Electron Microscope (H7700, Hitachi, Japan).

### ELISA analysis

Protein levels of IL-1β (#70-EK201B/3, Multisciences, China) and IL-18 (#70-EK282/4, Multisciences, China) in the supernatant of brain tissue homogenate and cell culture were measured and quantified using ELISA kits according to the manufacturer’s instructions. Absorbance at 450 nm was recorded, and the concentration of the target protein was quantified according to the standard curve. The result was expressed as pg/ml protein.

### Real-time polymerase chain reaction

Total RNA was extracted from the ipsilateral hemispheres of MCAO using TRIzol Reagent (Takara, Japan). And total RNA was reverse transcribed into cDNA with the PrimeScript RT reagent kit (Thermo Fisher Scientific, USA). Then, real-time PCR was performed with TB Green Premix Ex Taq II (Tli RNaseH Plus) using the Applied Biosystems 7500 Real-Time PCR System (Applied Biosystems, CA, USA) under the following conditions: predenaturation at 95 °C for 5 min, followed by 40 cycles at 95 °C for 15 s, 60 °C for 30 s, 95 °C for 15 s, 60 °C for 30 s, and 95 °C for 15 s. The relative mRNA expression level were normalized in relevance to β-actin. Primers used in the study are listed in Table 1.

**Table 1:** Primers used in real-time PCR

Gene	Sense primer (5′-3′)	Antisense primer (3′-5′)
ChemR23	AAGACCGTGAACACTGTGTGGTT	TAGGTGATGTGCATCGGCAA
NLRP1	CAGTTGCTCTTGAGATGGTCAG	ATCCAGTGAGGTTGGTGTTATTC
NLRP3	GACTGCGAGAGATTCTACAGC	CCTCCTCTTCCAGCAAATAGT
NLRC4	TTGTCACCACCACCACGGAG	ATCTTTGGCACTGTCTTCGGTCA
AIM2	AACTACCTTTGGCACAGTAACTT	GACTTCGTGGTTAGATTCAACA
β-actin	CCTCTATGCCAACACAGT	AGCCACCAATCCACACAG

### Western blot analysis

Brain tissues or treated cells were lysed with RIPA buffer supplemented with phosphatase and protease inhibitors and were then collected for protein extraction. The protein concentration was determined using a BCA kit. An equal amount of protein samples was loaded on sodium dodecyl sulfate (SDS)-polyacrylamide gel for electrophoresis at 120 V for 90 min. Subsequently, proteins were transblotted onto polyvinylidene fluoride (PVDF) membranes by wet transfer (400 mA, 90 min). The membrane was blocked by 5% non-fat milk in Tris-buffered saline with Tween-20 (TBST) for 2 h at room temperature and then incubated overnight at 4 °C with the primary antibodies (ChemR23, PA5-50932, 1:500, Invitrogen, USA; NLRP3, GB114320, 1:1000, Servicebio, China; apoptosis-associated speck-like protein containing a CARD (ASC), 10500-1-AP, 1:1000, Proteintech, IL, USA; Caspase-1 p20, AG-20B-0042, 1:1000, AdipoGen, Switzerland; GSDMD-N terminal (GSDMD-N), GB114198, 1:1000, Servicebio, China; tubluin, 11224-1-AP, 1:1000, Proteintech, IL, USA). After washing with TBST, the membranes were incubated with the secondary antibody (anti-rabbit and mouse IgG, 1: 5000; Santa Cruz Biotechnology, USA) for 2 h at room temperature, and then reacted with enhanced chemiluminescence (ECL, Invitrogen, USA). The result of chemiluminescence was recorded and analyzed with ImageJ software.

### Cell culture and ChemR23 overexpression

SH-SY5Y neuroblastoma cells purchased from Shanghai Institute of Cell Biology were cultured in Dulbecco’s modified Eagle medium (DMEM, Gibco, MD, USA) supplemented with 10% fetal bovine serum (FBS, Gibco, USA), 1% penicillin/streptomycin at 37 °C in an atmosphere containing 5% CO_2_.

A lentiviral vector overexpressing ChemR23 (OE) and a negative control vector (Lv-con) were purchased from Genomeditech (Shanghai, China). Stably transfected SH-SY5Y cells overexpressing ChemR23 were generated by using the overexpression plasmid vector PGMLV-CMV-MCS-3 × Flag-PGK-Puro. Briefly, ChemR23 (NCBI Gene ID: 14747) was amplified using the following primers: forward primer, 5′-GCGAATTCGAAGTATACCTCGAG-3′; reverse primer, 5′-GTCATGGTCTTTGTAGTCGGATCC-3′. The amplified sequences were inserted into PGMLV-CMV-MCS-3 × Flag-PGK-Puro according to the manufacturer’s instructions (Genomeditech, Shanghai, China). The constructed plasmid was added to 293 T cells. The supernatant containing the virus was collected at day 3. Then, the supernatant was used to infect the SH-SY5Y cells for 1 day. To obtain stably transfected lines, the cells were selected with 6 μg/ml puromycin. Finally, western blot analysis was used to assess the relative expression level of ChemR23 in transfected SH-SY5Y cells (Additional file 1: Fig. S2).

### Oxygen and glucose deprivation (OGD) modeling and drug treatment

For OGD modeling, SH-SY5Y were transferred in a glucose- and serum-free hypoxia chamber (STEMCELL Technologies Inc, USA) at 37 °C. The chamber was filled with premixed gas mixture of 1% O_2_, 5% CO_2,_ and 94% N_2_. To determine the appropriate degree of cell damage, the states of cells exposed to OGD were evaluated at different periods of time (for 1 h, 2 h, 4 h, 8 h, and 12 h). Control cells were maintained in a normoxic condition (5% CO_2_ and 95% air) with regular DMEM. For in vitro ChemR23 stimulation or inhibition assay, SH-SY5Y were pretreated with different doses of ChemR23 agonists (RvE1 or C-9) or inhibitor (α-NETA, No. GC45213, GLPBIO, USA) before being exposed to OGD. Cells in the ChemR23 agonists groups were cultured in a medium with different concentrations of RvE1 or C-9 (10, 100, 250, 500 or 1000 nM). In vitro NLRP3 inhibition experiments were conducted by pre-incubating the SH-SY5Y with 10 μM MCC950 (No. S8930, Selleck, USA) before OGD-induction. Doses of 10 μM α-NETA and 10 μM MCC950 were determined according to the previous study [22, 23].

### Cell viability

After OGD, SH-SY5Y cell viability were detected with the Cell Counting Kit-8 (CCK-8) (Dojindo, Japan) according to the manufacturer’s instructions. The absorbance of each well at 450 nm was obtained by an enzyme-labeled instrument. Relative cell viability was calculated as follows: (experimental group absorbance value/control group absorbance value) × 100%. Eight parallel wells were performed for each treatment group of cells, and the experiment was repeated for 3 times.

### Lactate dehydrogenase (LDH) release assay

The levels of LDH in the cell culture medium was measured by using an LDH kit according to the manufacturer’s instructions (Beyotime, China). LDH release (%) was calculated by calculating the ratio of experimental LDH level to control LDH level.

### Flow cytometry

SH-SY5Y cell death was determined using flow cytometry with an Annexin V-FITC/propidium iodide (PI) assay kit (Dojindo, Japan) according to the manufacturer’s instructions. Briefly, single-cell suspensions were stained with Annexin V-FITC (5 μL) and PI (5 μL) at room temperature for 15 min in darkness and were then detected by a flow cytometer (BD Biosciences, San Jose, CA) within 1 h. Data were analyzed using FlowJo.

### Scanning electron microscopy (SEM)

SH-SY5Y cells were fixed in 2.5% glutaraldehyde for 3 h and then rinsed with PBS. The cells were then dehydrated in ethanol (60%, 70%, 80%, 90%, and 99.9%, 30 min in each step). Ethanol was removed and dried with tert-butanol. Samples were stored at 4 °C until completely frozen, transferred to − 80 °C for 1 day, and subsequently freeze-dried. The samples were sputter-coated with a thin layer of gold before SEM analysis.

### Statistical analysis

Statistical Analysis among different groups was analyzed using one-way or two-way analysis of variance (ANOVA) with Tukey’s post hoc test or Student’s t test using GraphPad Prism 8.0 (GraphPad Software, Inc Inc., La Jolla, CA, USA). All data were presented as mean ± standard deviation (mean ± SD) and a statistical difference was set at P < 0.05. The statistical analyses were performed with GraphPad Prism 8 software.

## Results

### The time course of ChemR23 expression after MCAO

**Fig. 1:**
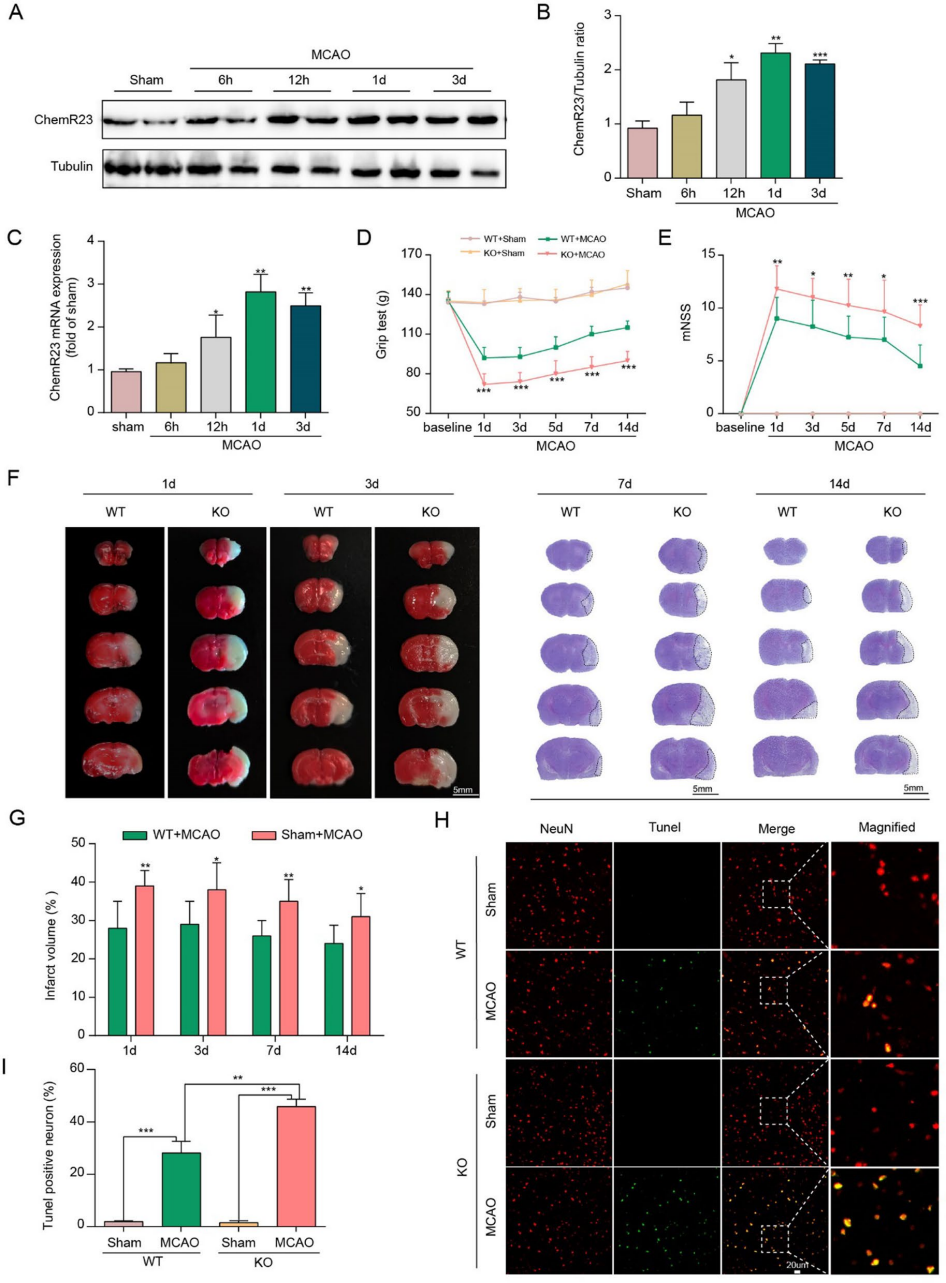
ChemR23 knockout aggravated post-stroke neurological deficits, infarct volume and neuronal cell death. **A**, **B** Representative immunoblots and quantification of ChemR23 in the ipsilateral brain at 6 h, 12 h, 1 day and 3 days after MCAO (n = 3/per group). **C** qRT-PCR analysis of ChemR23 in the ipsilateral brain at 6 h, 12 h, 1 day and 3 days after MCAO (n = 3/per group) (**D**, **E**) Grip test and mNSS assessment of mice in each group at Day 1, Day 3, Day 7, and Day 14 after MCAO (n = 10/per group). **F**, **G** Representative images of brain slices by TTC staining and Cresyl Violet staining in each group at Day 1, Day 3, Day 7, and Day 14 after MCAO and the quantitative analysis of infarct volume (n = 6/per group). Scale bar = 5 mm. **H**, **I** Representative immunofluorescence staining images and quantification of neuronal cell death based on NeuN and TUNEL assay in the ischemic ipsilateral brain regions at Day 1 after MCAO (n = 3/per group). Scale bar = 20 μm. Data are represented as mean ± SD. *P < 0.05, **P < 0.01, ***P < 0.001

Firstly, to determine the dynamic changes of ChemR23 expression after MCAO, we analyzed brain samples by qRT-PCR and western blot analysis in ipsilateral hemisphere with (6 h, 12 h, 1d and 3d after MCAO) and without MCAO. As shown in Fig. 1A–C, compared with Sham group, the expression of ChemR23 was marginally triggered at 12 h after MCAO surgery, peaked at Day 1 post-MCAO, and then gradually declined at Day 3, coinciding with similar tendency of ChemR23 levels from qRT-PCR. These data imply that cerebral ischemia triggers ChemR23 expression in the acute phase, and the pathophysiological function of ChemR23 needs to be further determined.

### ChemR23 deficiency exacerbated neurological deficits, infarct volume and neuronal death in MCAO mice

To determine how ChemR23 affected brain injury and neuronal cell death, we further utilized ChemR23 knockout mice to explore the function of ChemR23 in ischemic stroke. Neurological deficits were assessed via mNSS and grip strength test in all groups before or after MCAO. As shown in Fig. 1D and E, there was no obvious difference in performance of mNSS and grip strength test among the four groups before MCAO. Neurological impairment was observed in MCAO group compared to Sham group in WT mice. In ChemR23 KO mice, neurological impairment was further exacerbated as compared to the WT mice at Day 1, 3, 7, and 14 after MCAO, suggesting that ChemR23 deficiency exacerbates post-stroke neurological deficits. Then, we analyzed the infarct volume in the acute phase of cerebral infarction by TTC staining at Day 1 and Day 3 after operation, as well as in the chronic phase by Cresyl Violet staining at Day 7 and Day 14. As shown in Fig. 1F and G, the infarct volumes were remarkably enlarged by genetic ablation of ChemR23 compared with those in WT mice at Day 1, 3, 7 and 14 following MCAO. Additionally, we also observed that the body weight at Day 1 after MCAO was significantly decreased by ChemR23 deletion (Additional file 1: Fig. S3A). We then further explored the effect of ChemR23 deficiency on neuronal cell death, and found that ChemR23 deletion increased TUNEL-labeled cells at Day 1 after ischemic stroke (Fig. 1H and I). Collectively, these results indicate that ChemR23 knockout aggravates brain injury and neuronal cell death after ischemic stroke.

### ChemR23 deficiency amplified NLRP3 inflammasome activation in cerebral ischemia injury

**Fig. 2:**
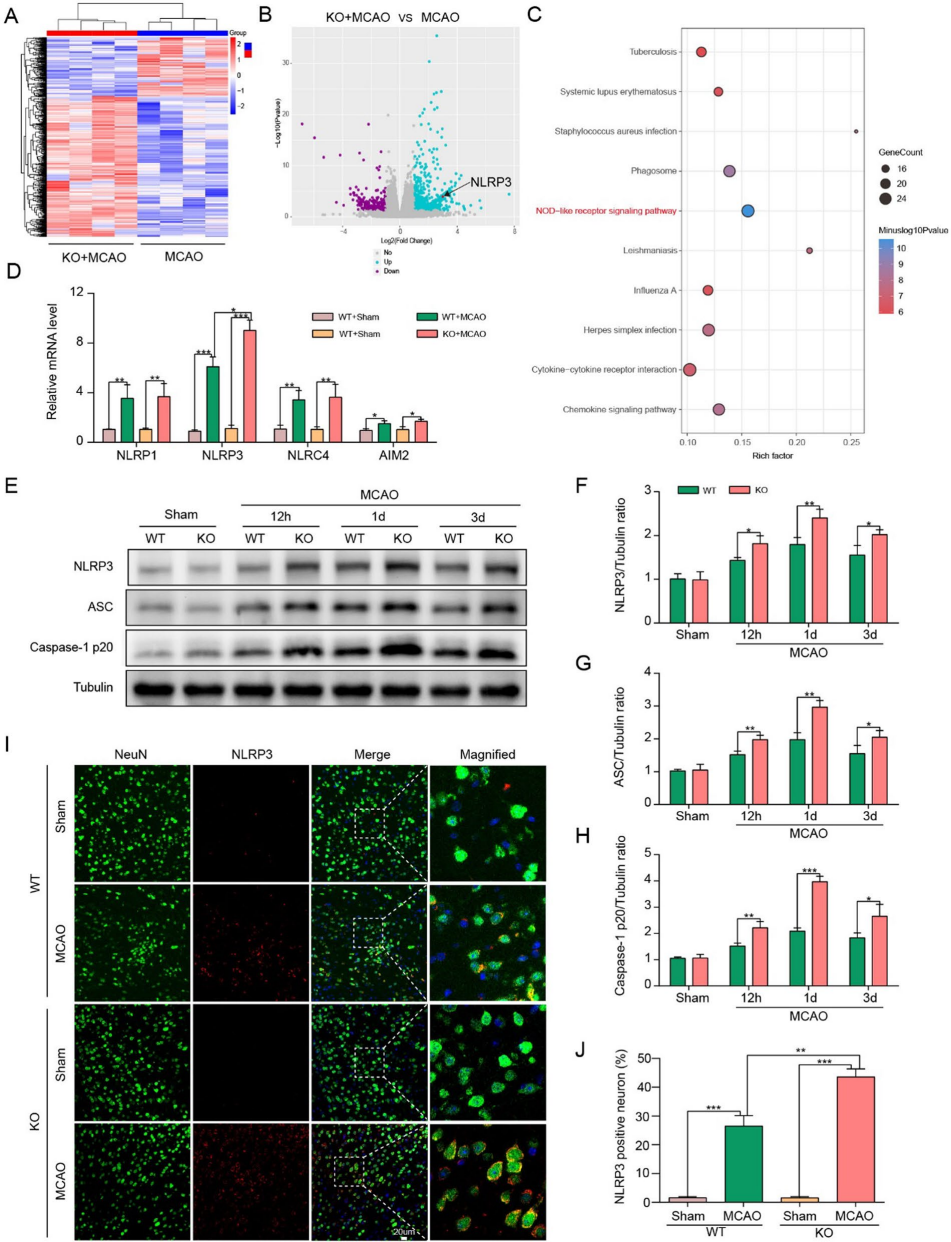
ChemR23 deletion amplified NLRP3 inflammasome activation in cerebral ischemia injury. **A**, **B** Thermograms and volcano plots showing the comparison between ChemR23 KO group and WT group at Day 1 after MCAO (n = 4/per group). **C** The KEGG pathway enrichment analysis (n = 4/per group). **D** qPCR analysis of the infammasome sensor genes NLRP1, NLRP3, NLRC4, and AIM2 ipsilateral hemispheres of WT mice and KO mice subjected to MCAO at Day 1 (n = 3/per group). **E**–**H** Western blotting and quantitative analysis of NLRP3, ASC, caspase-1 p20 expression in ischemic penumbra tissue at 12 h, Day 1 and Day 3 after MCAO (n = 3/per group). **I**, **J** Representative immunofluorescence staining images of NLRP3 were co-stained with NeuN in peri-infarct areas and their quantification at Day 1 after MCAO (n = 3/per group). Scale bar = 20 μm. Data are represented as mean ± SD. *P < 0.05, **P < 0.01, ***P < 0.001

To explore the underlying mechanisms of ChemR23 signaling in cerebral ischemia, we performed RNA-seq analysis of the ipsilateral hemispheres of MCAO at Day 1. As shown by the thermogram and volcano map (Fig. 2A, B), we found that 547 genes exhibited differentially expression (fold change > 2) in ChemR23 KO group, among which 518 genes were upregulated, and 29 genes were downregulated, indicating the expression profile of ChemR23 KO was distinctly different from that of WT group after MCAO. According to the KEGG pathway enrichment analysis (Fig. 2C), the NOD-like receptor signaling pathway stood out as the most affected pathway. Then we evaluated the expression of inflammasome sensor genes (i.e., NLRP1, NLRP3, NLRC4 and AIM2 inflammasomes) by qRT-PCR in ipsilateral hemispheres of WT mice and KO mice subjected to MCAO at Day 1. We found the increase of NLRP3 expression levels was the most significant among the inflammasome sensor genes analyzed (Fig. 2D). Consistently, accumulating evidence has shown that NLRP3 inflammasome signaling pathway is involved in ischemic stroke [8, 9]. Therefore, we sought to explore whether ChemR23 signaling works through NLRP3 inflammasome pathway in ischemic stroke.

The immunoblotting results illustrated that the expression of core NLRP3 inflammasome components, including NLRP3, ASC and caspase-1 p20, increased from 12 h post-MCAO surgery, plateaued at Day 1 post-MCAO, and then dropped at Day 3 post-MCAO. Meanwhile, compared with WT MCAO group, ChemR23 knockout further enhanced the increase of these proteins, especially at Day 1 post-MCAO (Fig. 2E–H). Based on these findings, Day 1 after MCAO was selected as the targeted time point for the subsequent experiments. To further explore the expression of NLRP3 in neurons, double immunofluorescence staining of NLRP3 /NeuN was performed on brain sections in peri-infarct regions. The results showed that ischemia-induced elevation of NLRP3 expression in neurons was prominently augmented by ChemR23 deletion (Fig. 2I, J). Taken together, our data indicate that ChemR23 knockout may aggravate brain injury by activating NLRP3 inflammasome.

### ChemR23 deficiency promoted neuronal pyroptosis induced by ischemic stroke

**Fig. 3:**
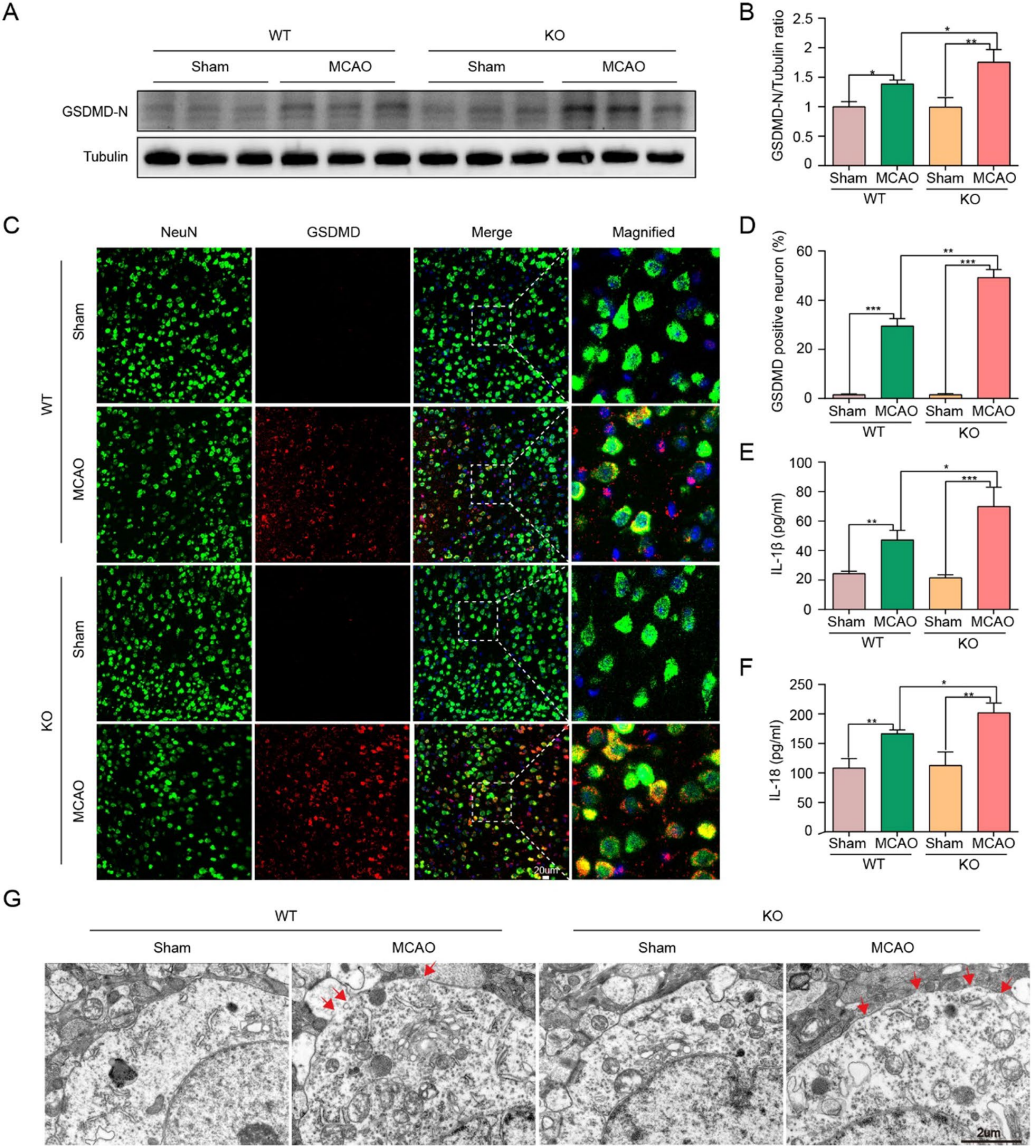
ChemR23 deficiency promoted neuronal pyroptosis in MCAO mice. **A**, **B** Western blot and quantitative analysis of GSDMD-N at Day 1 after MCAO (n = 3/per group). **C**, **D** Double immunostaining of GSDMD with NeuN in ischemic penumbra region and quantitative analysis at Day 1 after MCAO (n = 3/per group). Scale bar = 20 μm. **E**, **F** ELISA analysis for IL-1β and IL-18 levels in ipsilateral brain tissues (n = 4/per group). **G** Representative transmission electron microscopy pictures of neurons in ischemic penumbra. Pores on neuronal membrane are pointed out with red arrows (n = 3/per group). Scale bar = 2 μm. Data are represented as mean ± SD. *P < 0.05, **P < 0.01, ***P < 0.001

**Fig. 4:**
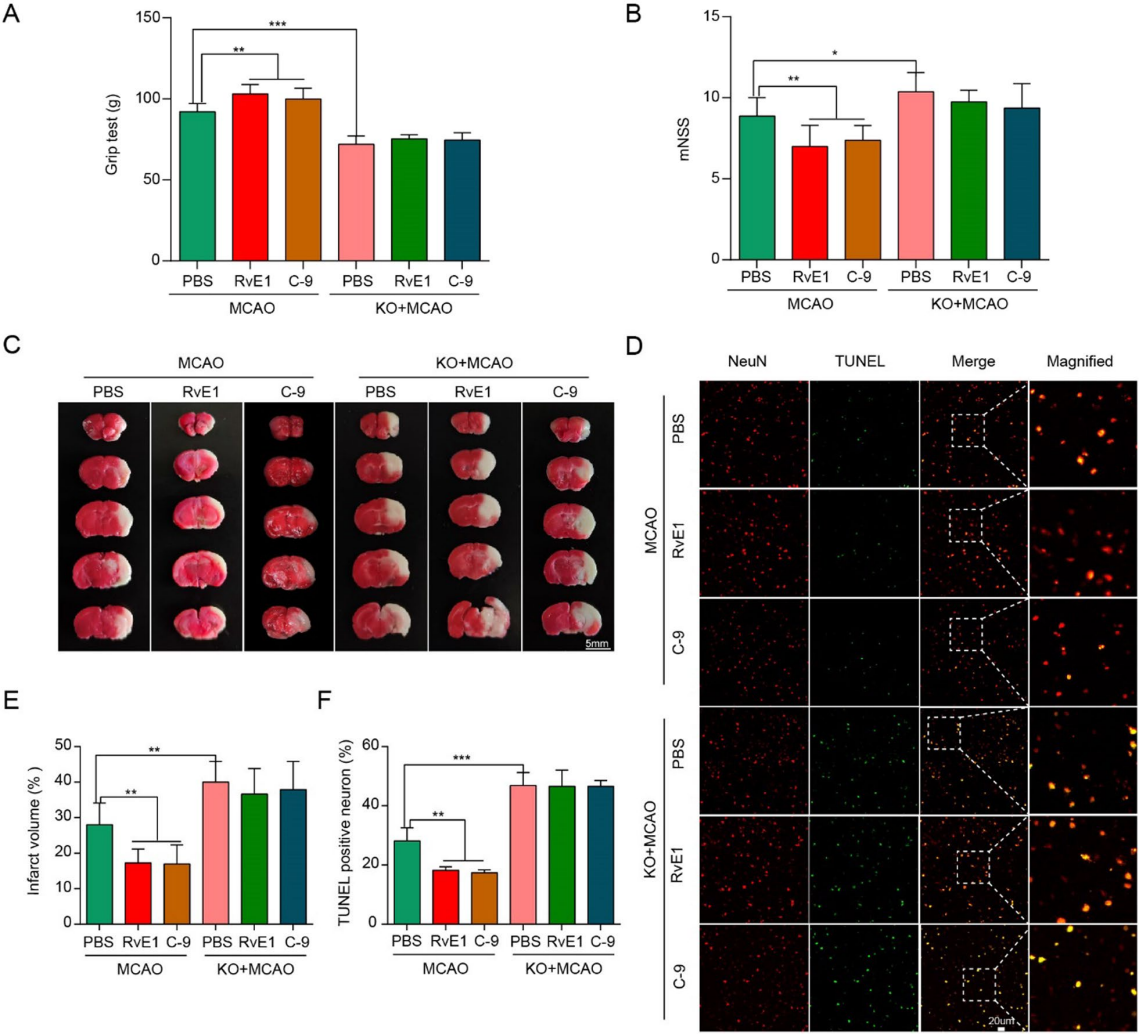
RvE1 and C-9 improved neurological deficits, infarct volumes and neuronal death after MCAO. **A**, **B** Grip test and mNSS assessment of mice in each group (n = 10/per group). **C**, **E** TTC-stained sections in each group and the quantitative analysis of infarct volume at Day 1 after MCAO (n = 6/per group). Scale bar = 5 mm. **D**, **F** Representative immunofluorescent images and quantification of neuronal death by NeuN and TUNEL staining in the ischemic ipsilateral brain regions at Day 1 after MCAO (n = 3/per group). Scale bar = 20 μm. Data are represented as mean ± SD. *P < 0.05, **P < 0.01, ***P < 0.001

Upon activation, NLRP3 inflammasome promotes the cleavage of pro-caspase-1 to caspase-1 p20, which then cleaves the pyroptotic factor GSDMD and inherently leads to pyroptosis. Next, we investigated whether ChemR23 could affect neuronal pyroptosis in MCAO model. Western blot analysis demonstrated cerebral ischemia increased the levels of GSDMD-N (the activated form of GSDMD), and the increase of GSDMD-N was notably boosted by ChemR23 deletion in MCAO mice (Fig. 3A, B). Consistently, to further explore the expression levels of GSDMD in neurons, double immunofluorescence staining of GSDMD /NeuN was performed on brain sections in peri-infarct regions. Intensive GSDMD immunostaining accumulating on the neuronal membrane was observed in MCAO mice, forming a “ring of fire” morphology (Fig. 3C). GSDMD/NeuN double immunofluorescent labeling indicated that more GSDMD-positive neurons were observed in ischemic penumbra region in MCAO group compared to Sham group, and this trend was evidently exaggerated in ChemR23 knockout mice after MCAO modeling (Fig. 3C, D). Subsequently, ELISA was used to further evaluate the expression of pyroptosis-related proteins in the ischemic penumbra. As presented in Fig. 4E and F, the levels of IL-1β and IL-18 in ChemR23 deficiency mice were obviously increased when compared with WT mice after MCAO. The increase of these pro-inflammatory cytokines is also an important sign of pyroptosis pathophysiology induced by caspase-1 cleavage and NLRP3 inflammasome activation [8]. In addition, TEM was employed to observe pores formed by GSDMD-N on neuronal cell membrane after MCAO. As shown in Fig. 4G, the GSDMD pores in neurons at the ischemic penumbra region were more frequent in ChemR23 KO mice compared to WT mice after MCAO. Therefore, we provided substantial evidence that MCAO triggers pyroptotic cell death in neurons, and further confirmed the important role of ChemR23 deficiency in promoting MCAO-induced neuronal pyroptosis.

### Activation of ChemR23 by RvE1 or C-9 attenuated neurological deficits and brain injury in MCAO mouse model

As we observed significant deleterious effects of ChemR23 knockout in cerebral ischemia, we then investigated whether the activation of ChemR23 by RvE1 or C-9 could ameliorate the brain injuries. Different dosages of RvE1 (0.3 and 1.0 nmol) and C-9 (3, 9 and 27 μg/kg) were administrated intracerebroventricularly 30 min before MCAO. Neurological deficits and infarct volume were measured at Day 1 after MCAO. As depicted in Additional file 1: Fig. S4, RvE1 i.c.v. dose-dependently improved neurological deficits and infarct volume in the WT MCAO group. For C-9, dosage of 3 μg/kg did not affect neurological deficits and infarct volume, while higher doses of C-9 (9 and 27 μg/kg) significantly attenuated neurological deficits and decreased infarct volume. Furthermore, RvE1 or C-9 treatment could both rescue weight loss after MCAO (Additional file 1: Fig. S3B). According to the results above, 1.0 nmol of RvE1 and 27 μg/kg of C-9 were selected for further experiments.

Compared to WT MCAO mice, RvE1 and C-9 treatment notably improved neurological performance (shown by mNSS and grip strength test) and decreased infarct volume at Day 1 after MCAO (Fig. 4A–E). On the other hand, TUNEL staining further revealed that RvE1 and C-9 ameliorated neuronal cell death (Fig. 4D, F). Intriguingly, administration with either RvE1 or C-9 in ChemR23 knockout mice could not rescue the neurological deficits, weight loss, infarct volume, or neuronal cell death (Fig. 4A–E), indicating that the beneficial effects of RvE1 and C-9 are ChemR23-dependent. Overall, these results indicate that activation of ChemR23 signaling by RvE1 or C-9 could attenuate neurological deficits and brain injury in ischemic stroke.

### Activation of ChemR23 signaling ameliorated brain injury via NLRP3 inflammasome-mediated neuronal pyroptosis after MCAO

**Fig. 5:**
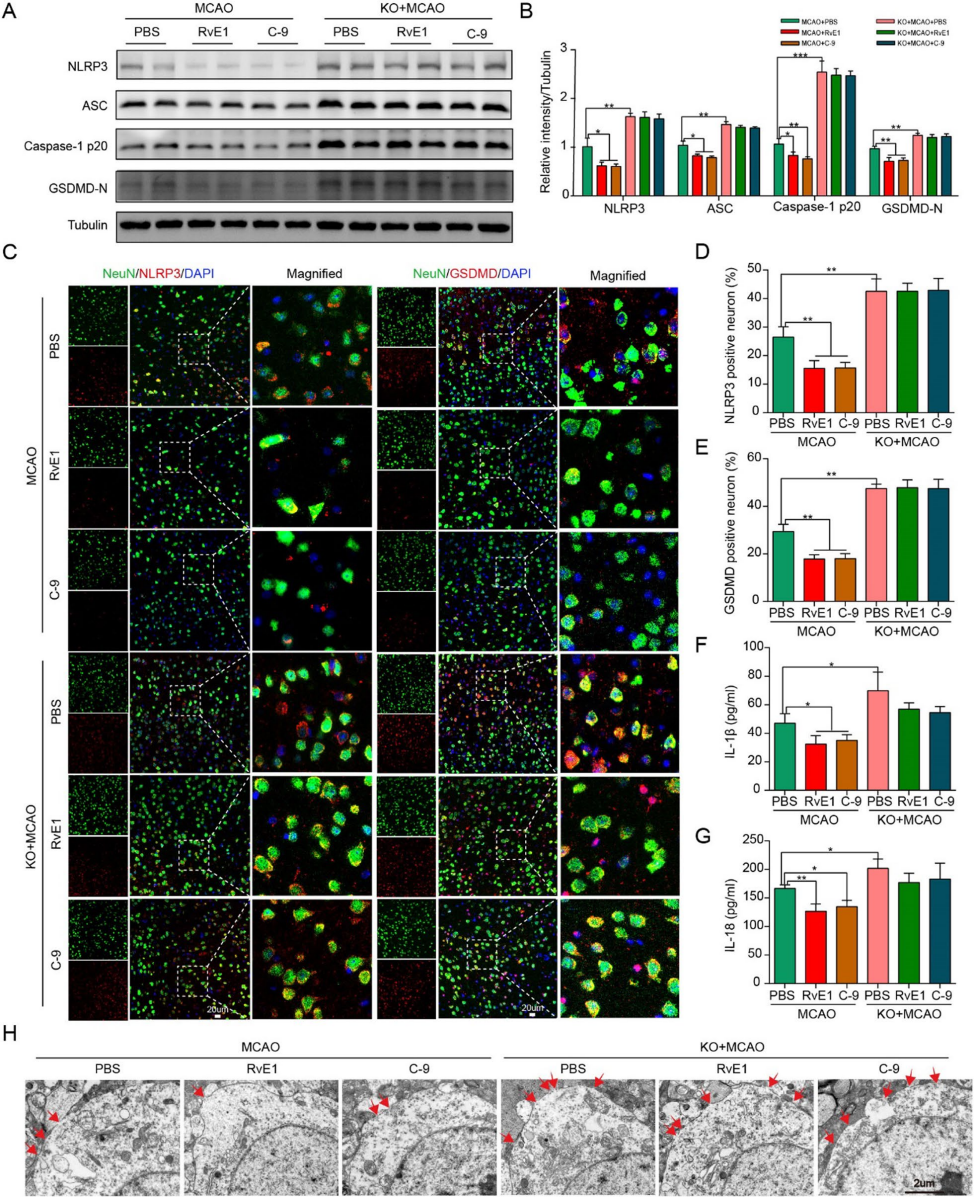
RvE1 and C-9 ameliorated NLRP3 inflammasome-mediated pyroptosis in MCAO mice. **A**, **B** Immunoblots and quantitative analysis of NLRP3, ASC, caspase-1 p20, GSDMD-N (n = 3/per group). **C**–**E** Double immunofluorescent staining of NLRP3 or GSDMD with NeuN in ischemic penumbra region and quantitative analysis at Day 1 after MCAO (n = 3/per group). Scale bar = 20 μm. **F**, **G** The ELISA assays for IL-1β and IL-18 levels in ipsilateral brain tissues subjected to ischemia at Day 1 (n = 4/per group). **H** Representative microphotographs and quantification of neuronal death based on NeuN and TUNEL assay in the ischemic ipsilateral brain regions at Day 1 after MCAO (n = 3/per group). Scale bar = 2 μm. Data are represented as mean ± SD. *P < 0.05, **P < 0.01, ***P < 0.001

As mentioned above, ChemR23 deficiency was able to aggravate brain injury and activate NLRP3 inflammasome-mediated neuronal pyroptosis after MCAO. Next, we further sought to explore whether ChemR23 activation by RvE1 and C-9 could ameliorate NLRP3 inflammasome-mediated pyroptosis. Subsequently, western blot, double immunofluorescence staining and ELISA were used to evaluate the expression of pyroptosis-related proteins. As demonstrated in Fig. 5A and B, there was a significant decrease in the expressions of NLRP3, ASC, caspase-1 p20, and GSDMD-N after RvE1 and C-9 treatment in WT MCAO mice, and these beneficial effects were absent in ChemR23 KO mice. Besides, double immunofluorescence staining of NLRP3/NeuN and GSDMD/NeuN was performed on ischemic penumbra. The results suggested that administration of ChemR23 agonists of RvE1 or C-9 in WT MCAO mice significantly prevented the increase in NLRP3 positive neurons and GSDMD positive neurons. Yet, ChemR23 knockout abolished these beneficial effects (Fig. 5C–E). Subsequently, ELISA assays revealed comparable findings of reduced levels of IL-1β and IL-18 in RvE1 or C-9 treated mice (Fig. 5F, G). In accordance with these findings, the GSDMD membrane pores in the ischemic penumbra region were markedly decreased by RvE1 or C-9 treatment. Furthermore, neither RvE1 nor C-9 could reverse the increase of GSDMD membrane pores in ChemR23 knockout mice (Fig. 5H). Based on the above results, we could conclude that ChemR23 activation ameliorates ischemic brain injury via inhibiting NLRP3 inflammasome-mediated pyroptosis.

### ChemR23 activation attenuated OGD-induced neuronal pyroptosis.

**Fig. 6:**
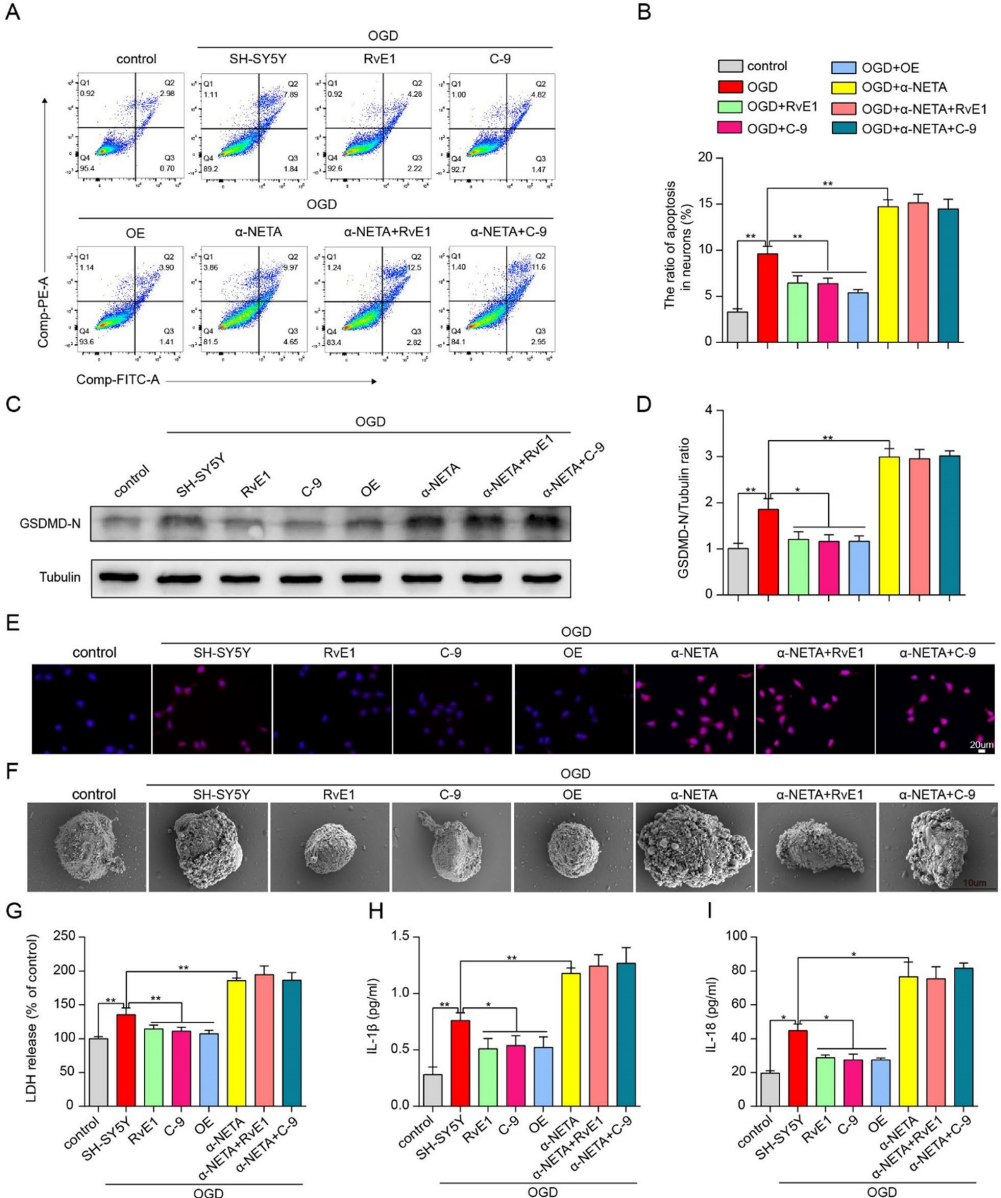
ChemR23 activation attenuated OGD-induced neuronal pyroptosis. **A**, **B** Dead neurons were detected by Annexin V/PI kit 4 h after OGD. **C**, **D** Western blotting and quantitative analysis of GSDMD-N in treated neurons after OGD. **E** Representative immunofluorescent images of GSDMD in cultured neurons. Scale bar = 20 μm. **F** Representative scanning electron microscopy pictures of neurons showing morphological changes of pyroptosis induced by OGD. Scale bar = 10 μm. **G** LDH release was assessed and quantified in neurons subjected to OGD for 4 h. **H**, **I** ELISA assays for IL-1β and IL-18 levels in supernatants of neuron subjected to OGD for 4 h. At least three independent experiments were repeated. Data are represented as mean ± SD

To confirm the protective effects of ChemR23 activation in SH-SY5Y cells, an experimental model of OGD with 1% oxygen was established to mimic MCAO in vitro. To determine the optimal time point in OGD model, CCK-8 analysis was performed to analyze cell viability. As shown in Additional file 1: Fig. S4A, compared with the control group, the cell viability gradually decreased with OGD duration, and had a reduction of 28.91% at OGD 4 h. Hence, we chose the optimal time point of OGD as 4 h. Then, the optimal dose of RvE1 and C-9 was determined, and a CCK-8 assay was used to assess cell viability. After OGD, cell viability was significantly reduced, while RvE1 or C-9 remarkably reversed the reduction (Additional file 1: Fig. S4B). RvE1 or C-9 at 500 nM were selected for the subsequent experiments (Additional file 1: Fig. S4B). Subsequently, SH-SY5Y cells were genetically overexpressed with ChemR23 and pharmacologically subjected to ChemR23 antagonist α-NETA to investigate the effects of activation or inhibition of ChemR23 signaling. The Annexin V-FITC/PI assay showed that cell death decreased significantly with ChemR23 agonists RvE1 or C-9 treatment and ChemR23 overexpression, but increased significantly with ChemR23 inhibitor α-NETA under OGD conditions (Fig. 6A, B). Concomitantly with the observation in vivo, both western blot and immunostaining results showed that elevated GSDMD-N under OGD was relieved by RvE1 or C-9 or overexpression of ChemR23, but was further exacerbated by α-NETA (Fig. 6C–E and Additional file 1: Fig.S6B-D). It is well-known that GSDMD-N form membrane pores, followed by rapid membrane rupture. LDH has been reported to be an indicator of cell membrane permeability [24], therefore, the cytotoxic effect was further measured by LDH assay. As expected, we found that OGD considerably increased the release of LDH into cell culture supernatant, which was exacerbated by α-NETA administration. However, treatment with RvE1 or C-9 and ChemR23 overexpression attenuated OGD-induced LDH release (Fig. 6G and Additional file 1: Fig.S6A). As pyroptosis is characterized by cellular swelling and the emergence of large bubbles from the plasma membrane [25], we further observed the morphological characteristics of pyroptosis by using SEM. In the representative images of SEM, cellular swelling and the emergence of large bubbles from the plasma membrane under OGD were improved by RvE1 or C-9 or overexpression of ChemR23 but were further exacerbated by α-NETA (Fig. 6F). Furthermore, ELISA assay was performed to evaluate the levels of pro-inflammatory cytokines (including IL-1β and IL-18) in the cell culture supernatant associated with neuronal pyroptosis. Compared to the control group, OGD-induced SH-SY5Y cells expressed remarkably higher levels of IL-1β and IL-18, which was reversed by RvE1 or C-9 treatment or ChemR23 overexpression. These effects of RvE1 or C-9 treatment was abrogated by α-NETA treatment (Fig. 6H, I), suggesting that the neuroprotective of RvE1 and C-9 is ChemR23-dependent. Collectively, these data indicated that ChemR23 activation improves while ChemR23 inhibition promotes OGD-induced neuronal pyroptosis.

### The neuroprotective effect of ChemR23 against OGD-induced neuronal pyroptosis was NLRP3 dependent.

**Fig. 7:**
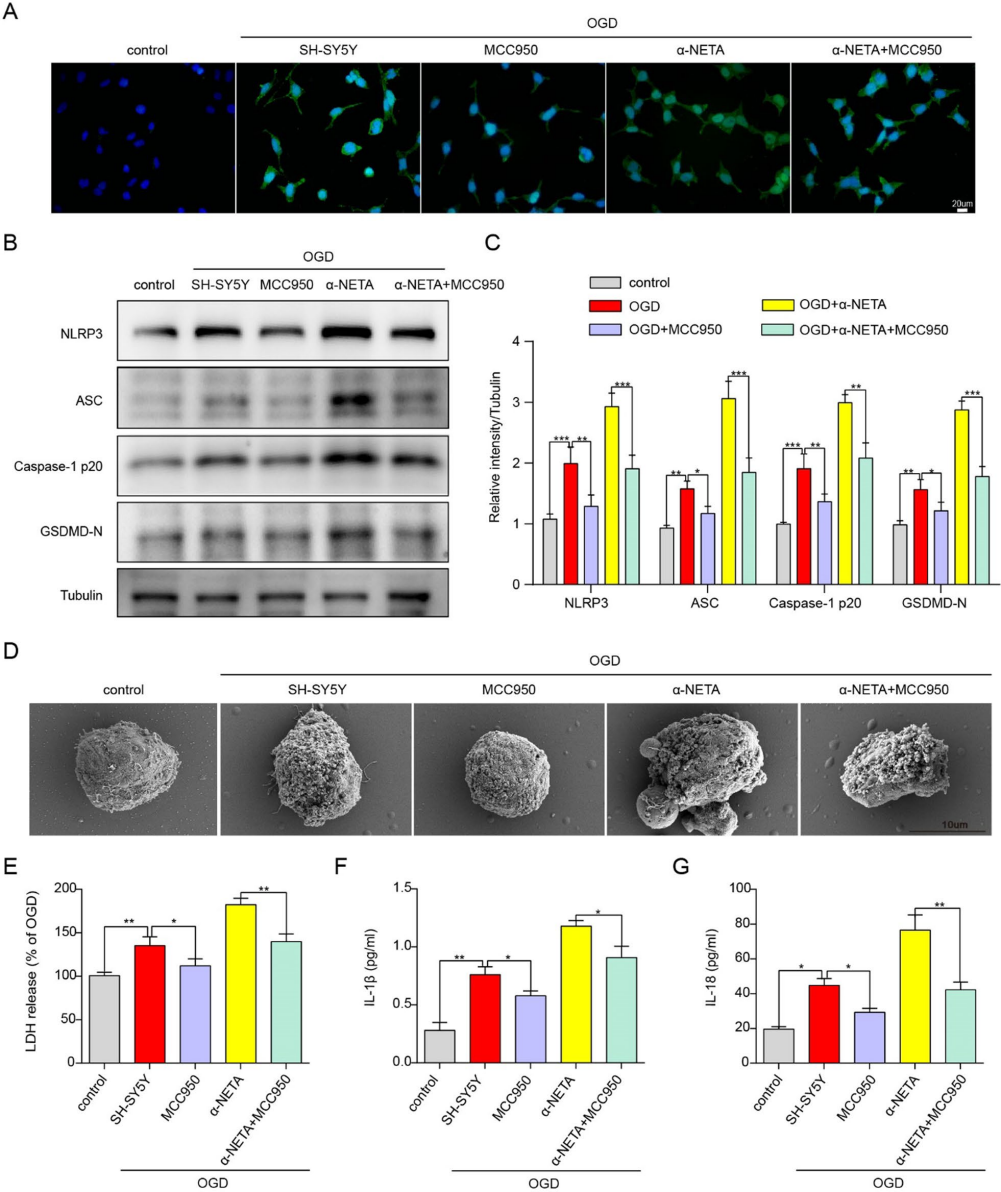
The neuroprotective effect of ChemR23 against OGD-induced neuronal pyroptosis was NLRP3 dependent. **A** Representative immunofluorescent images of NLRP3 in cultured neurons. Scale bar = 20 μm. **B**, **C** Immunoblots and quantitative analysis of NLRP3, ASC, caspase-1 p20, GSDMD-N in treated neurons after OGD. **D** Representative scanning electron microscopy pictures of neurons. Scale bar = 10 μm. **E** LDH release was assessed and quantified in neurons subjected to OGD for 4 h. **F**, **G** ELISA analysis for IL-1β and IL-18 levels in supernatants of neuron subjected to OGD for 4 h. At least three independent experiments were repeated. Data are represented as mean ± SD. *P < 0.05, **P < 0.01, ***P < 0.001

To further explore whether ChemR23 signaling regulated neuronal pyroptosis via NLRP3 inflammasome pathway in vitro, SH-SY5Y cells were exposed to the NLRP3 inflammasome inhibitor MCC950 under OGD condition. The results of immunofluorescent staining showed that α-NETA markedly increased NLRP3 expression in OGD-induced SH-SY5Y cells, whereas the fluorescent intensities of NLRP3 in SH-SY5Y cells co-culture with α-NETA and MCC950 were much lower (Fig. 7A). We further evaluated the protein levels of the key components of NLRP3 inflammasome signaling by Western blotting. As presented in Fig. 7B and C, the protein levels of NLRP3, ASC, Caspase-1 p20, and GSDMD-N were remarkably increased after α-NETA administration, however, co-culture α-NETA with MCC950 reversed these changes. Similarly, α-NETA administration increased OGD-induced SH-SY5Y cell membrane permeability, as indicated by significantly increased LDH release and the appearance of large bubbles on the plasma membrane, which could be reversed when OGD-induced SH-SY5Y cells were co-cultured with α-NETA and MCC950 (Fig. 7D, E). The secretion of IL-1β and IL-18 was further examined by ELISA assay. As shown in Fig. 7F and G, α-NETA led to a remarkable induction of IL-1β and IL-18, whereas co-cultured α-NETA with MCC950 reversed ChemR23-inhibition-mediated up-regulation of IL-1β and IL-18. Taken together, these in vitro findings indicated that ChemR23 inhibition aggravates OGD-induced pyroptosis by activating NLRP3 inflammasome.

## Discussion

**Fig. 8:**
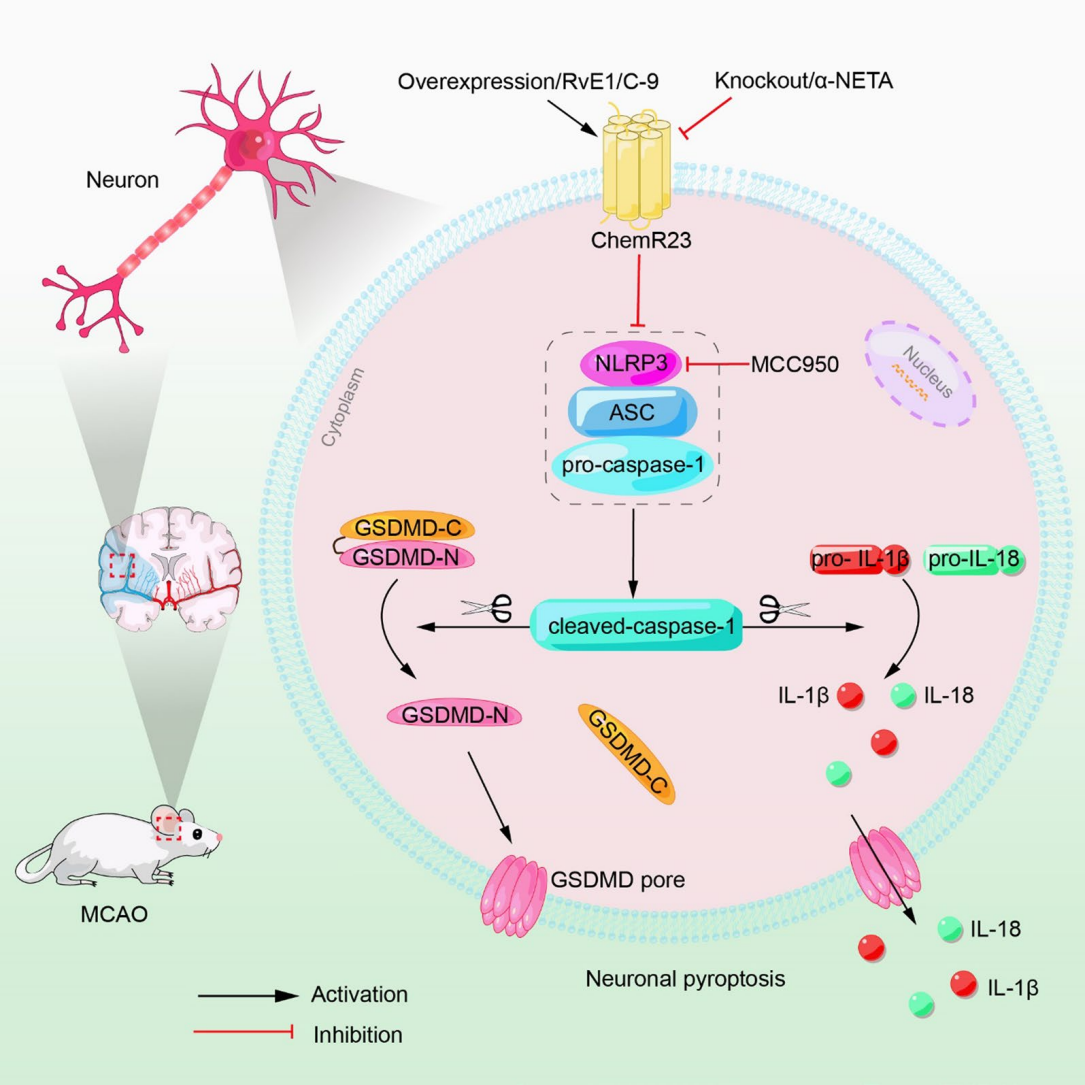
Schematic diagram for the mechanisms of ChemR23 signaling in neuronal pyroptosis following cerebral ischemia. ChemR23 knockout or inhibition triggers NLRP3-ASC-caspase-1 inflammasome activation, leading to increased inflammatory cytokines IL-18 and IL-1β. The triggered caspase-1 cleaves GSDMD to promote the release of N-terminal domain, which further executes pore formation on the neuronal membrane. On the other hand, ChemR23 agonists (RvE1 or C-9) or overexpression of ChemR23 can reverse the above molecular pathway abnormalities

The present study demonstrated that ChemR23 signaling regulates NLRP3 inflammasome-mediated neuronal pyroptosis in ischemic stroke. We observed that the expression of ChemR23 was upregulated following acute cerebral ischemia in MCAO mice. ChemR23 knockout aggravated neuronal pyroptosis and inflammatory response by provoking NLRP3 inflammasome activation and recruitment, leading to enlarged cerebral infarct volumes and exacerbated neurological impairments. On the other hand, ChemR23 activation by RvE1 or C-9 could remarkably reduce NLRP3 inflammasome-mediated neuronal pyroptosis in MCAO mice (Fig. 8). Furthermore, by using in vitro model, we also demonstrated that ChemR23 signaling protected neuronal cells from NLRP3 inflammasome-mediated pyroptosis under OGD condition. Overall, our data for the first time revealed the critical role of ChemR23 signaling in ischemic stroke.

ChemR23 is a chemoattractant receptor that exerts various biological effects depending on the binding of different ligands [10, 26]. RvE1, derived from ω-3 polyunsaturated fatty acid eicosapentaenoic acid (EPA) [27], interacted with ChemR23 to reduce infarct size and accelerate cardiac recovery through inhibiting inflammatory cell recruitment and cardiomyocyte apoptosis [14]. Chemerin, another ligand of ChemR23, has been reported to improve infarct size, and neurological impairment by alleviating neuroinflammation and neuronal injury via ChemR23 after stroke or neonatal hypoxic-ischemic encephalopathy [17, 28]. These previous reports indicated a potential role of ChemR23 signaling in ischemic diseases. In the present study, we reported for the first time that the expression of ChemR23 in the brain is elevated within Day 3 post stroke. This phenomenon further guided us to investigate the effects of ChemR23 knockout in MCAO mice. Compared with WT MCAO mice, ChemR23 knockout mice developed severer post-stroke neurological deficits and exhibited larger infarct volumes. Thus, the post-stroke elevation of ChemR23 may be a protective feedback mechanism against cerebral ischemia.

ChemR23 can be activated by either RvE1 or chemerin. RvE1, a bioactive lipid mediator derived from EPA, is a potent agonist of ChemR23 [26]. Chemerin-9 (C-9), a nonapeptide corresponding to the C terminus of the processed form of human chemerin, is also a strong agonist for ChemR23 [29]. In addition to ChemR23, RvE1 and C-9 have been observed to interact with other receptors such as leukotriene B4 receptor 1 (BLT1) and chemokine CC motif receptor-like 2 (CCRL2), respectively [30, 31]. We showed that administration of C-9 or RvE1 in WT MCAO mice could ameliorate neurological deficits, reduce infarct volume, and decrease neuronal cell death. These protective effects were abolished in ChemR23 knockout mice. Thus, our results showed that the beneficial effects of RvE1 and C-9 were ChemR23-dependent but not through BLT1 or CCRL2. Combined with the ChemR23 knockout data, we could conclude that the activation of ChemR23 signaling is beneficial and inhibition of ChemR23 signaling is harmful following cerebral ischemia.

The molecular mechanisms of ChemR23 signaling in ischemic stroke are of further interest. By RNA-seq analysis in the ipsilateral hemispheres of MCAO mice, we found that NOD-like receptor signaling pathway was the most overactivated pathway in ChemR23 knockout mice. Among multiple inflammasomes, the NLRP3 inflammasome is the most typical one [5]. Increasing evidence has implicated that activation of NLRP3 inflammasome in ischemic stroke regulates neuroinflammation and neuronal cell death, while inhibition of NLRP3 attenuates neurological deficits and infarct volumes [8, 32]. Here, we showed that the levels of NLRP3 were significantly increased both in vivo in MCAO mice and in vitro in OGD model in neurons, as evidenced by western blot and immunofluorescence staining of NLRP3. Genetic knockout of ChemR23 in mice or pharmacological inhibition of ChemR23 in neurons further increased the levels of NLRP3 inflammasome. Thus, ChemR23 signaling may regulate NLRP3 inflammasome pathway in ischemic stroke. To further confirm this, we demonstrated that ChemR23 activation by RvE1 or C-9, or overexpression of ChemR23, all could alleviate neuronal cell death and NLRP3 inflammasome activation, which related to ameliorated neurological deficits and infarct volumes. Yet, knockout of ChemR23 abolished the effects of RvE1 and C-9 on NLRP3 inflammasome. These data further demonstrated that RvE1 and C-9 regulate NLRP3 inflammasome to benefit ischemic stroke via ChemR23. Subsequently, we showed that the increased cell death in ChemR23-inhibited neurons was reversed by treatment with MCC950, the inhibitor of NLRP3 inflammasome. Our findings are in line with recent studies that NLRP3 deficiency or inhibition was associated with reduced infarct volume and improvement of neurological outcomes [32, 33]. Thus, our results elucidated that ChemR23 signaling regulates NLRP3-inflammasome activation in ischemic stroke.

Upon the activation of NLRP3 inflammasome, NLRP3 will assemble with ASC to induce the cleavage and activation of pro-caspase-1, which subsequently cleaves pro-IL-1β and pro-IL-18 into biologically mature IL-1β and mature IL-18 [34, 35]. We showed that the levels of IL-1β and IL-18 were drastically upregulated after MCAO in mice and OGD in neurons. These increased pro-inflammatory cytokines can lead to further neuroinflammation and neural injury [8]. Activated caspase-1 also mediates the cleavage of GSDMD to form 12–14 nm membrane pores [7]. Thereafter, these membrane pores dissipate cellular ionic gradients, which then cause water influx, DNA fragmentation, rapid plasma membrane rupture, cell swelling, and eventual osmotic lysis combined with the release of inflammatory factors [36]. Such a process is defined as a special type of cell death, namely pyroptosis. Pyroptosis has been demonstrated to play a crucial role in brain injury after cerebral ischemia [8, 37]. In line with these reports, we found upregulated expression of GSDMD-N was coupled with formation of membrane pores both in vivo after MCAO and in vitro following OGD. We further confirmed that ChemR23 knockout or inhibition led to enhanced GSDMD-N expression, elevated IL-1β and IL-18 levels, and increased membrane pores and large bubbles in neurons. Collectively, we show for the first time that ChemR23 signaling plays a crucial role in pyroptosis in ischemic stroke. Furthermore, ChemR23 activation by RvE1 and C-9, or overexpression of ChemR23 notably diminished GSDMD-N expression, downregulated IL-1β and IL-18 levels, and decreased membrane pores and large bubbles in neurons. These effects were abolished by ChemR23 knockout and mimicked by MCC950, the inhibitor of NLRP3 inflammasome. Accordingly, these data indicated that activation of ChemR23 signaling is neural protective against NLRP3 inflammasome-mediated pyroptosis in ischemic stroke.

## Conclusions

In summary, our study provides novel evidence that ChemR23 signaling could ameliorate brain injury via inhibiting NLRP3 inflammasome-mediated pyroptosis in cerebral ischemia. Our findings highlighted the critical role of ChemR23 in ischemic stroke, and targeting ChemR23 may be a promising therapeutic method therein.

### Supplementary Information

**Additional file 1: Fig. S1** Experimental designs in animal models. (A) The study design of the expression pattern of ChemR23 after MCAO. (B) The study design about the effects of ChemR23 deficiency on ischemic stroke. (C) The study design analyzing the effects of activating ChemR23 by RvE1 and C-9 on ischemic stroke. **Fig. S2** Representative immunoblots and quantification of ChemR23 overexpression in SH-SY5Y cells. At least three independent experiments were repeated. Data are represented as mean ± SD. *P < 0.05, **P < 0.01, ***P < 0.001. **Fig. S3** Weight changes among the groups before and after MCAO at Day 1. n = 8 per group. Data are represented as mean ± SD. *P < 0.05, **P < 0.01, ***P < 0.001. **Fig. S4** RvE1 and C-9 treatment improved neurological deficits and infarct volumes after MCAO in a dose-dependent manner. (A, B) Grip test and mNSS assessment of mice in each group (n = 10/per group). (C, D) TTC-stained sections in each group and the quantitative analysis of infarct volume at Day 1 after MCAO (n = 6/per group). bar =5 mm. Data are represented as mean ±SD. Compared with MCAO group *P < 0.05, **P < 0.01, ***P < 0.001. **Fig. S5** Cell viability of SH-SY5Y was measured after OGD. (A) Cell viability of SH-SY5Y was measured with CCK8 assay at different time points of OGD. (B) Cell viability was measured in RvE1 or C-9 treatment group at distinct concentrations at 4 h post-OGD. **Fig. S6** ChemR23 activation attenuated OGD-induced GSDMD-N expression. (A, B) Western blotting and quantitative analysis of GSDMD-N in neurons after OGD. (E) Representative immunofluorescent images of GSDMD in cultured neurons. Scale bar = 20 μm. At least three independent experiments were repeated. Data are represented as mean ± SD. *P < 0.05, **P < 0.01, ***P < 0.001.

## Data Availability

All data generated or analyzed during this work are available upon request.
