# Supplementary material for: ChemR23 signaling ameliorates brain injury via inhibiting NLRP3 inflammasome-mediated neuronal pyroptosis in ischemic stroke
Source: J Transl Med. 2024 Jan 4;22:23. doi: 10.1186/s12967-023-04813-0 (PMC10768115; doi:10.1186/s12967-023-04813-0)
Supplement: Supplementary file 1 — Additional file 1: Fig. S1 Experimental designs in animal models. (A) The study design of the expression pattern of ChemR23 after MCAO. (B) The study design about the effects of ChemR23 deficiency on ischemic stroke. (C) The study design analyzing the effects of activating ChemR23 by RvE1 and C-9 on ischemic stroke. Fig. S2 Representative immunoblots and quantification of ChemR23 overexpression in SH-SY5Y cells. At least three independent experiments were repeated. Data are represented as mean ± SD. *P < 0.05, **P < 0.01, ***P < 0.001. Fig. S3 Weight changes among the groups before and after MCAO at Day 1. n = 8 per group. Data are represented as mean ± SD. *P < 0.05, **P < 0.01, ***P < 0.001. Fig. S4 RvE1 and C-9 treatment improved neurological deficits and infarct volumes after MCAO in a dose-dependent manner. (A, B) Grip test and mNSS assessment of mice in each group (n = 10/per group). (C, D) TTC-stained sections in each group and the quantitative analysis of infarct volume at Day 1 after MCAO (n = 6/per group). bar =5 mm. Data are represented as mean ±SD. Compared with MCAO group *P < 0.05, **P < 0.01, ***P < 0.001. Fig. S5 Cell viability of SH-SY5Y was measured after OGD. (A) Cell viability of SH-SY5Y was measured with CCK8 assay at different time points of OGD. (B) Cell viability was measured in RvE1 or C-9 treatment group at distinct concentrations at 4 h post-OGD. Fig. S6 ChemR23 activation attenuated OGD-induced GSDMD-N expression. (A, B) Western blotting and quantitative analysis of GSDMD-N in neurons after OGD. (E) Representative immunofluorescent images of GSDMD in cultured neurons. Scale bar = 20 μm. At least three independent experiments were repeated. Data are represented as mean ± SD. *P < 0.05, **P < 0.01, ***P < 0.001. [file 12967_2023_4813_MOESM1_ESM.docx]

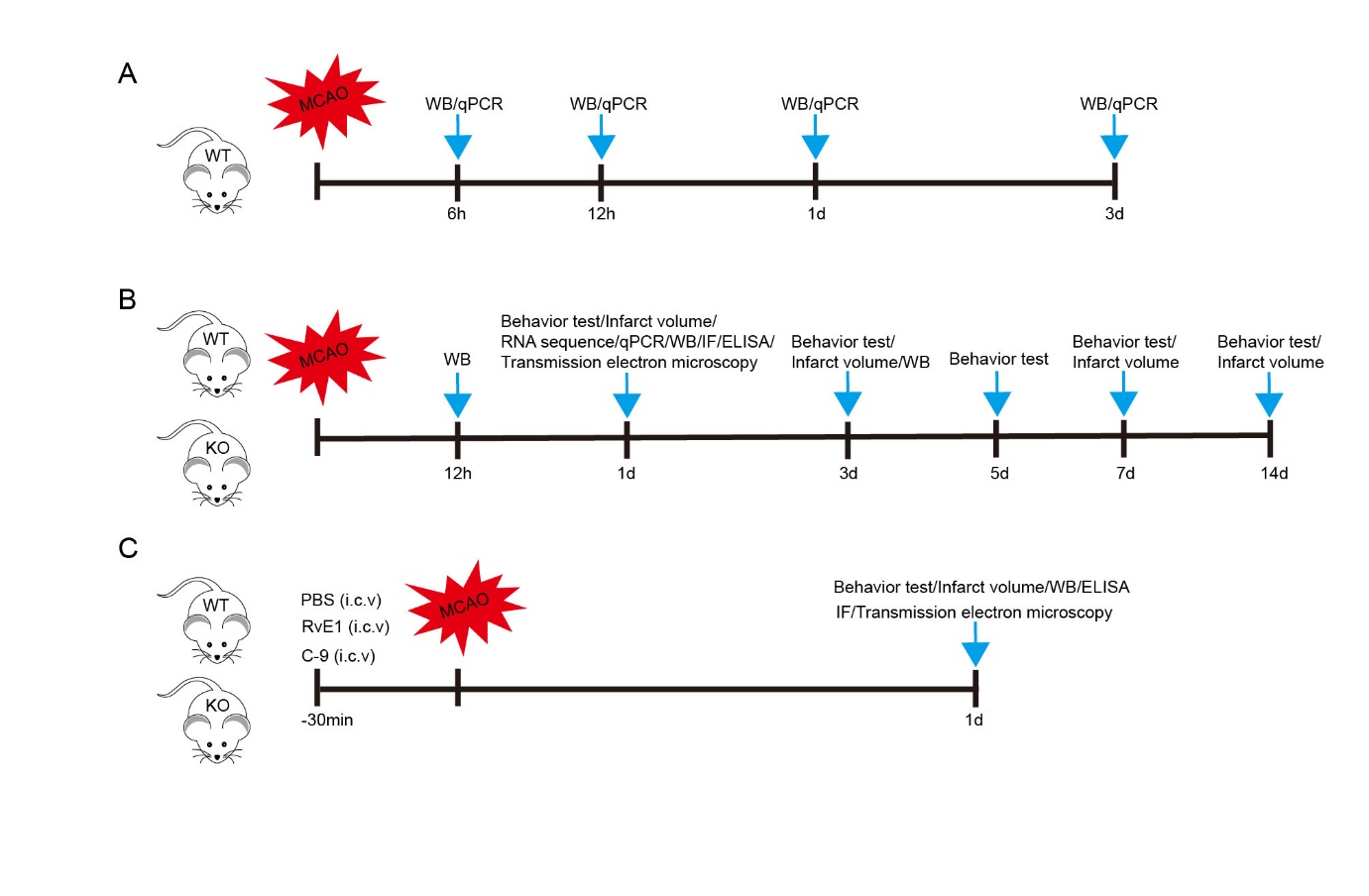


**Fig. S1 Experimental designs in animal models.** (A) The study design of the expression pattern of ChemR23 after MCAO. (B) The study design about the effects of ChemR23 deficiency on ischemic stroke. (C) The study design analyzing the effects of activating ChemR23 by RvE1 and C-9 on ischemic stroke.


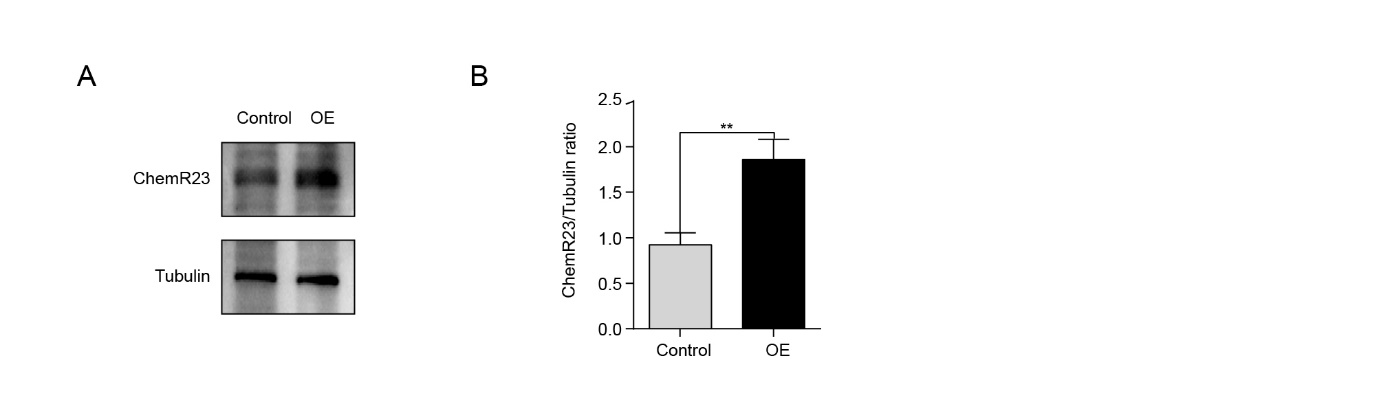


**Fig. S2 Representative immunoblots and quantification of ChemR23 overexpression in SH-SY5Y cells.** At least three independent experiments were repeated. Data are represented as mean ± SD. *P < 0.05, **P < 0.01, ***P < 0.001


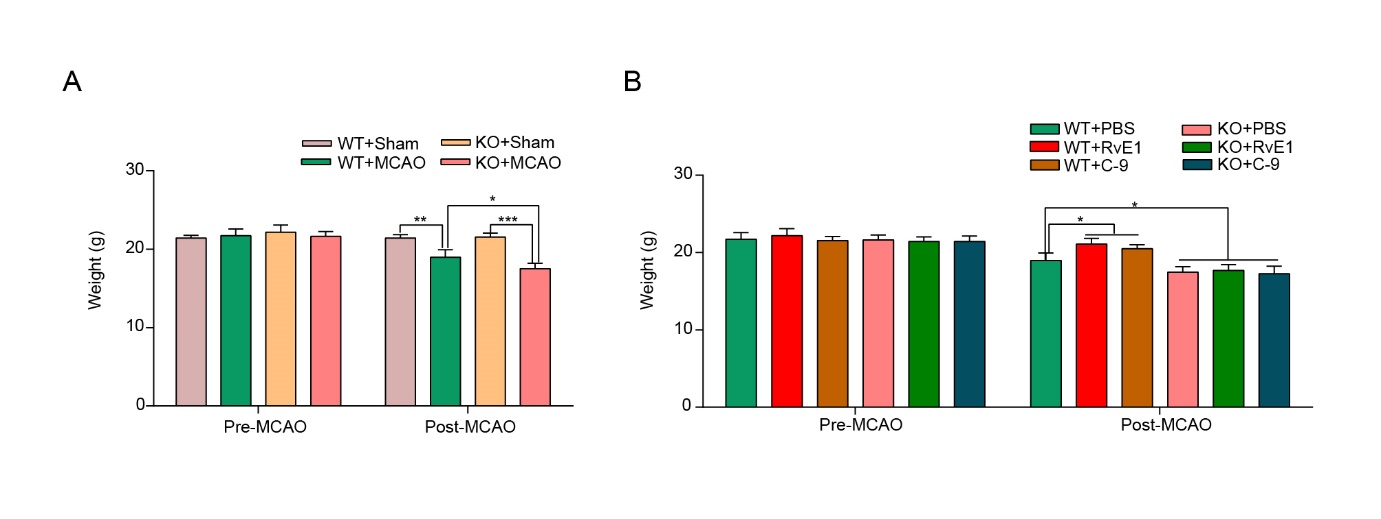


**Fig. S3 Weight changes among the groups before and after MCAO at Day 1.** n = 8 per group. Data are represented as mean ± SD. *P < 0.05, **P < 0.01, ***P < 0.001


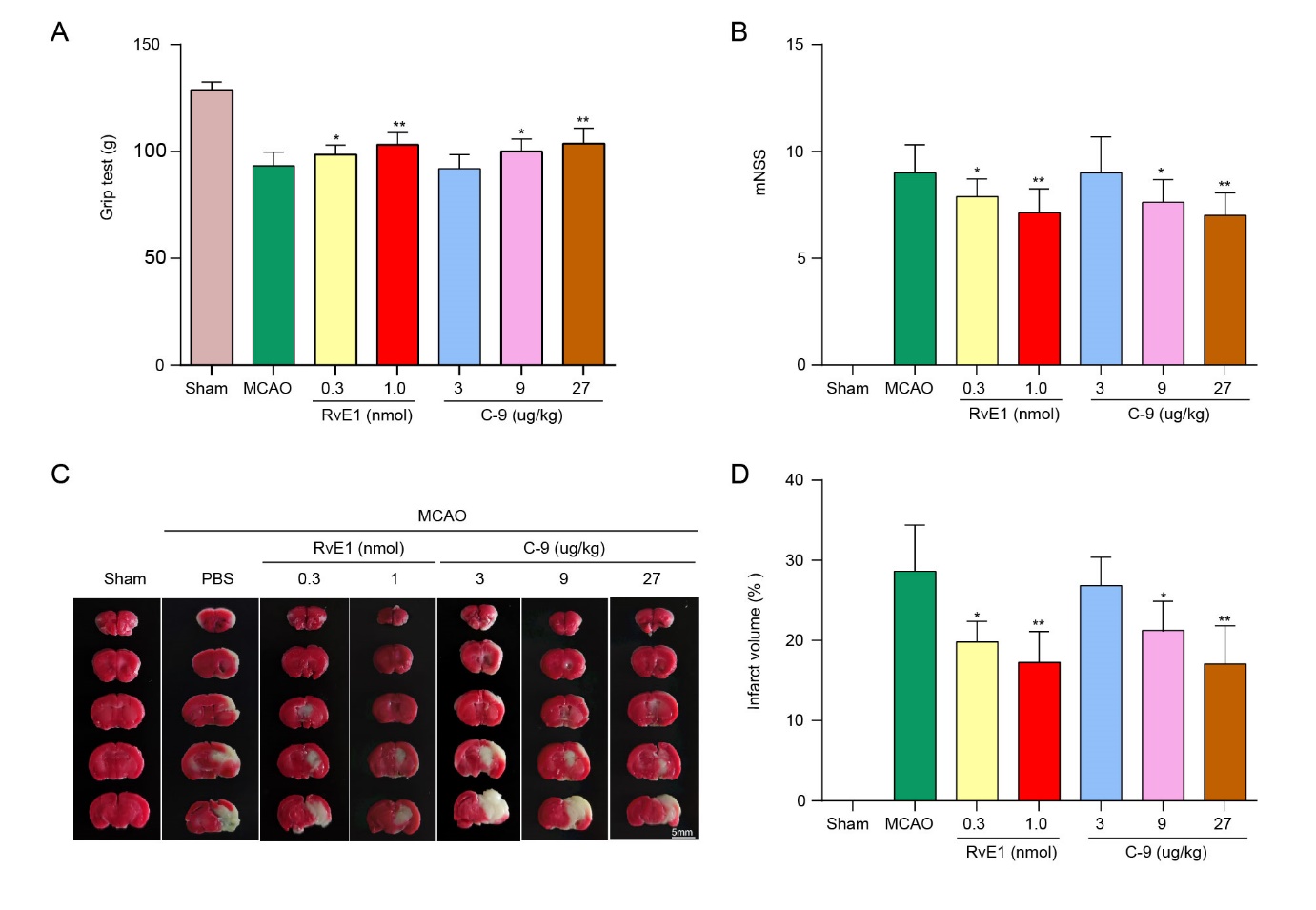


**Fig. S4 RvE1 and C-9 treatment improved neurological deficits and infarct volumes after MCAO in a dose-dependent manner.** (A, B) Grip test and mNSS assessment of mice in each group (n = 10/per group). (C, D) TTC-stained sections in each group and the quantitative analysis of infarct volume at Day 1 after MCAO (n = 6/per group). bar =5 mm. Data are represented as mean ±SD. Compared with MCAO group *P < 0.05, **P < 0.01, ***P < 0.001


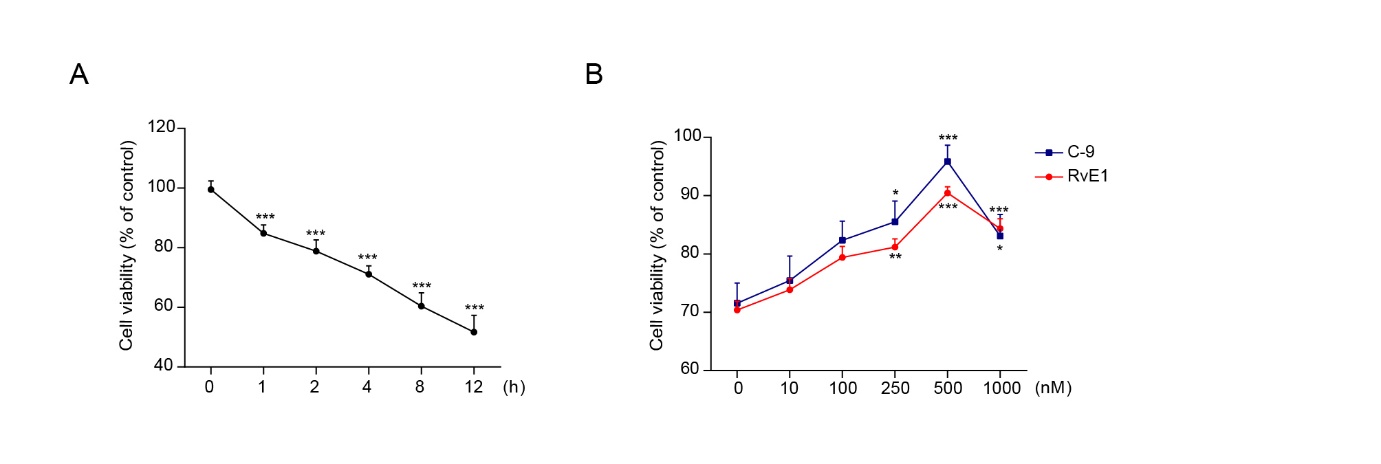


**Fig. S5 Cell viability of SH-SY5Y was measured after OGD.** (A) Cell viability of SH-SY5Y was measured with CCK8 assay at different time points of OGD. (B) Cell viability was measured in RvE1 or C-9 treatment group at distinct concentrations at 4 h post-OGD.


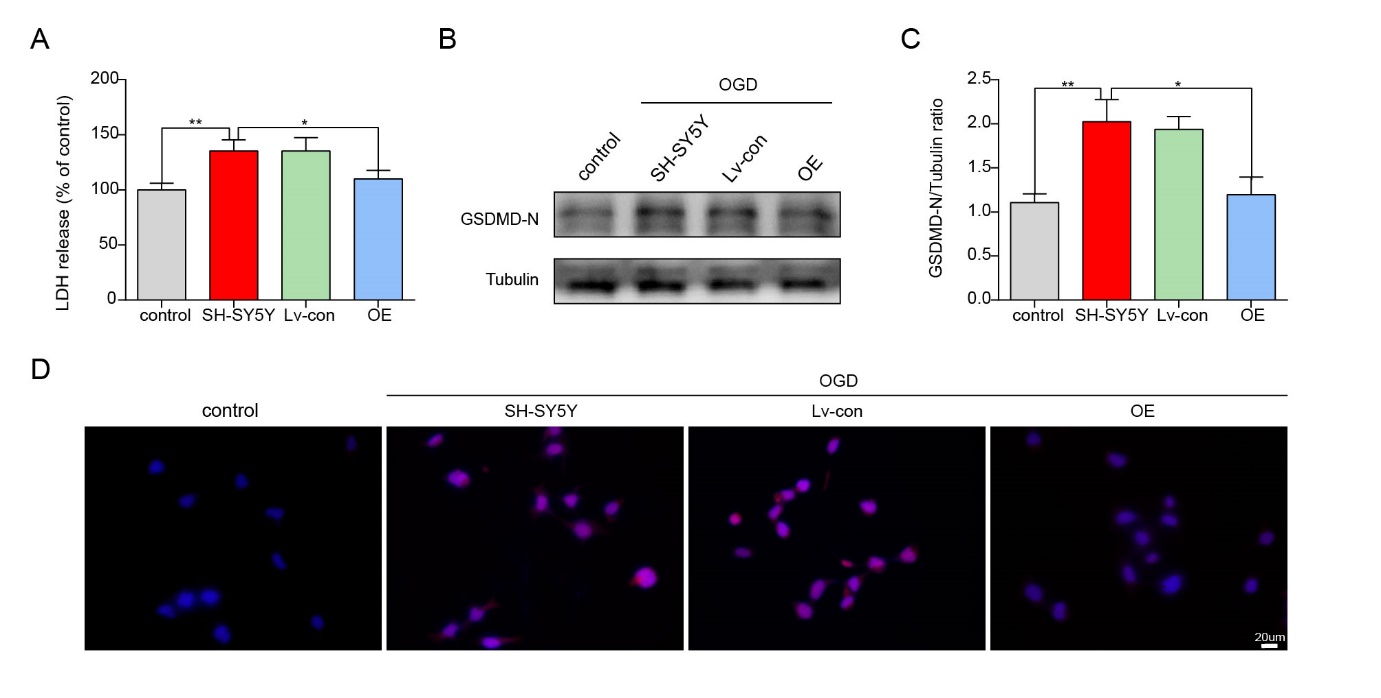


**Fig. S6 ChemR23 activation attenuated OGD-induced GSDMD-N expression.** (A, B) Western blotting and quantitative analysis of GSDMD-N in neurons after OGD. (E) Representative immunofluorescent images of GSDMD in cultured neurons. Scale bar = 20 μm. At least three independent experiments were repeated. Data are represented as mean ± SD. *P < 0.05, **P < 0.01, ***P < 0.001
